# Season and host-community composition inside roosts may affect host-specificity of bat flies

**DOI:** 10.1038/s41598-024-54143-4

**Published:** 2024-02-19

**Authors:** Attila D. Sándor, Alexandra Corduneanu, Sándor Hornok, Andrei D. Mihalca, Áron Péter

**Affiliations:** 1 HUN-REN-UVMB Climate Change: New Blood-Sucking Parasites and Vector-Borne Pathogens Research Group, Budapest, Hungary; 2 Department of Parasitology and Zoology, University of Veterinary Medicine, Budapest, Hungary; 3 Faculty of Veterinary Medicine, Department of Parasitology and Parasitic Diseases, University of Agricultural Sciences and Veterinary Medicine of Cluj-Napoca, Cluj-Napoca, Romania; 4 Department of Animal Breeding and Animal Production, University of Agricultural Sciences and Veterinary Medicine, Cluj-Napoca, Romania

**Keywords:** Ecology, Behavioural ecology

## Abstract

Bat flies are one of the most abundant ectoparasites of bats, showing remarkable morphological adaptations to the parasitic habit, while the relationship with their hosts is characterized by a high level of specificity. By collecting bat flies from live hosts, our intention was to elucidate the seasonal differences in bat fly occurrence and to describe factors regulating the level of incipient host specificity. Our results indicate that the prevalence and the intensity of infestation is increasing from spring to autumn for most host species, with significant differences among different fly species. Males showed higher infestation levels than females in autumn, suggesting a non-random host choice by flies, targeting the most active host sex. Bat-bat fly host specificity shows seasonal changes and host choice of bat flies are affected by the seasonal differences in hosts’ behavior and ecology, the intensity of infestation and the species composition of the local host community. Nycteribiid bat flies showed lower host specificity in the swarming (boreal autumn) period, with higher prevalence recorded on non-primary hosts. Choosing a non-primary bat host may be an adaptive choice for bat flies in the host’s mating period, thus increasing their dispersive ability in a high activity phase of their hosts.

## Introduction

Bats are the second most speciose group of mammals with more than 1400 species, with a distribution covering most continents and almost every habitat type. They are morphologically diverse, using very different dietary niches from insect hunting to feeding on fruits, pollen or blood. A series of characters make them unique among mammals, due to their ability of active flight, their ability to navigate with ultrasound, social roosting and their extreme long relative life-span^1^. Most species evolved into highly social systems due their requirement of safe roosting during the day. In this way, multiple individuals have to share relatively small spaces, forming dense colonies^2^. It is widely accepted that host ecology and behavior affect parasites and host coloniality enhances access to resources for most parasites. Therefore, bats are common and also frequent hosts for several ectoparasite species belonging diverse groups, like mites, ticks, bugs, fleas or flies^3,4^.

The most abundant ectoparasites of bats are the bat flies (Diptera: Nycteribiidae and Streblidae), mostly wingless flies, living in the fur and on the wing membranes and feeding with blood^5^. Although the earliest studies described them as generalist species (able to infest multiple host species^6^, later it was demonstrated that they infest host species very specifically^4,7^. Bat flies show a remarkable level of adaptation in morphology and behavior in accordance to their parasitic lifestyle to counteract the defense strategies of bats. There are around 570 bat fly species worldwide of which 17 are present in Europe, with 10 species reported until now both from Romania, as well Bulgaria^8–10^.

Parasites and their hosts have a very close relationship both in the present and over their evolutionary history^11^. One of the most important features of a parasite-host relationship is the level of specificity which determines how many species are used as hosts by a parasite. Host specificity may be determined by multiple factors, such as the morphological, physiological or behavioral characters of both the host and the parasite, as well the momentary state of the continuous arms-race between host and parasite over their long and shared evolutionary history^12^. Inside the bat-bat fly parasitic system there are multiple factors acting against a high-level specificity, like dispersal capacity or host searching time^13^. Polyxenous (multiple-host) parasites may increase their dispersal capacity (even if they are not able to fly, the majority of the bat fly species can easily switch hosts in communal roosts, they usually leave the host regularly during their lives, mostly because of their reproductive system—the female can spend one third of her life span off-host for larviposition^4,14^). Young adult bat flies (after their emergence from the puparium) may reduce the time of host searching if they are able to choose from multiple bat host species. However, this time is usually short, as several bat species tend to roost together in multiple types of shelters and they have all the chance to interact and share parasites with other species by roosting close to each other or by using the same underground passages^15^. In addition, using multiple host species may enable easier colonization of new areas and faster dispersal^13^. Against all odds, host specificity among bat flies is high, higher than in most ectoparasite species^15^. This observed high specificity of bat flies can be the result of simultaneous action of several major driving forces (intrinsic or acting from the hosts’ side), like the higher chances of mate availability on the primary host, by the lower immune, and behavioural response from the host (a milder induced grooming behavior from the host)^15,16^.

Bats may be reservoirs for a number of zoonotic diseases which can affect humans, livestock or companion animals and some of these are vector-borne. Bat flies are vectors for several pathogens (some even zoonotic), including bacteria, such as *Bartonella* spp.^10^, piroplasms^17^ or trypanosomes^18^. The epidemiologic importance of a competent vector is directly linked to its capacity to infest as many as possible new hosts (including different host species), thus host specificity is a key character of insect vectors. High host specificity is developed by a parasite to increase its ability to exploit a particular susceptible host, and typically originates from a long-term host-parasite co-speciation. As host specificity is a major factor influencing the circulation of pathogens in host-parasite systems, knowledge on host selection by bat flies may inform us on likeliness of pathogen spread by these vectors. Our knowledge on European bat ectoparasites is limited, although it was shown that bat flies may exert some impact on the ecology of the hosts^19,20^ and may be vectors of zoonotic diseases^10,21–24^.

As with most external parasites of bats, nycteribiid flies show differences in abundance according to sampling site^10^, season^25^, environment^26^, host species^9^, or the life-history stage of the host itself^25^. Bats roosting in underground shelters show higher general ectoparasite infestation levels^17,27^, with nycteribiid flies showing significantly higher prevalence as well intensity on cave-dwelling hosts^10^. Underground roosts—opposite to crevices—may provide bats (and their parasites) with a more stable environment (less fluctuations in thermal and hydrologic conditions, reduced disturbance), but are also characterized by larger and more loosely structured, multi-species host groups, occasional higher density of bats^1,28^. We suggest that host population structuring (species composition, seasonal dynamics and activity patterns) inside underground roosts has a major impact on the host-parasite relationships between individual bat and bat fly species. Using a dataset collected from cave-dwelling bats of SE Europe, hereby we would like to test two hypotheses: (1) we suggest that increase in fly abundance may trigger increased dispersion towards non-specific hosts and that (2) higher host diversity in the swarming period may lead to local or seasonal differences in host choice (and ultimately also host specificity) inside underground roosts, suggesting a more dynamic host-choice in dipteran ectoparasites of bats.

## Results

We collected a total of 6207 bat flies (ten species) from 5773 sampled bats, belonging to 23 species sampled at 64 individual sites (Table 1., On-line supplementary Table S1, S2). In addition, nine more bat species did not harbor any bat flies (*Eptesicus nilssoni* n = 3, *Hypsugo savii* n = 14, *Myotis davidii* n = 14, *Nyctalus lasiopterus* n = 1, *N. leisleri* n = 1, *Pipistrellus kuhlii* n = 1, *Pi. pipistrellus* n = 103, *Pi. pygmaeus* n = 8 and *Vespertilio murinus* n = 190). Data from these hosts was excluded from further analysis.

**Table 1:** Bat host species, number of captured individuals, number of bat fly species registered and number of individual bat flies collected (*number of primary bat fly species in parenthesis).

Bat species	*n*	Infested	Number of bat flies	Number of bat fly species*	Prevalence (%, CI 95%)	Infestation intensity (CI 95%)
*Miniopterus schreibersii*	2112	1355	3072	9 (2)	64.16 (62.07–66.21)	2.27 (2.16–2.37)
*Rhinolophus ferrumequinum*	502	117	196	8 (1)	23.31 (19.68–27.26)	1.68 (1.4–1.95)
*Myotis blythii*	389	225	496	8 (3)	57.84 (52.76–62.8)	2.2 (1.98–2.45)
*Myotis myotis*	316	197	566	5 (4)	62.34 (56.75–67.7)	2.87 (2.46–3.29)
*Myotis daubentonii*	266	122	286	7 (3)	45.86 (39.76–52.06)	2.34 (1.98–2.71)
*Myotis emarginatus*	248	4	9	3 (2)	1.61 (0.44–4.08)	2.25 (0.76–5.26)
*Rhinolophus euryale*	247	141	348	4 (1)	57.09 (50.66–63.34)	2.47 (2.06–2.88)
*Myotis capaccinii*	199	151	809	6 (2)	75.88 (69.32–81.65)	5.36 (4.69–6.02)
*Myotis nattereri*	158	24	46	5 (2)	15.19 (9.98–21.75)	1.92 (1.26–2.57)
*Nyctalus noctula*	154	2	5	1 (1)	1.3 (0.16–4.61)	2.5 (NA)
*Rhinolophus hipposideros*	151	4	4	2 (1)	2.65 (0.73–6.64)	1 (NA)
*Barbastella barbastellus*	124	4	5	2 (2)	3.23 (0.89–8.05)	1.25 (0.45–2.05)
*Rhinolophus mehelyi*	124	43	72	2 (1)	34.68 (26.36–43.75)	1.67 (1.43–1.92)
*Myotis brandtii*	110	3	3	2 (2)	2.73 (0.57–7.76)	1 (NA)
*Myotis bechsteinii*	104	55	135	1 (1)	52.88 (42.85–62.75)	2.45 (1.93–2.98)
*Plecotus auritus*	53	1	1	1 (1)	1.89 (0.05–10.07)	1 (NA)
*Pipistrellus nathusii*	39	1	2	2 (2)	2.56 (0.06–13.48)	2 (NA)
*Myotis mystacinus*	36	4	5	1 (1)	11.11 (3.11–26.06)	1.25 (0.45–2.05)
*Rhinolophus blasii*	33	12	25	1 (1)	36.36 (20.4–54.88)	2.08 (1.21–2.96)
*Eptesicus serotinus*	30	2	2	2 (2)	6.67 (0.82–22.07)	1 (NA)
*Plecotus austriacus*	28	1	1	1 (1)	3.57 (0.09–18.35)	1 (NA)
*Myotis alcathoe*	19	9	12	2 (2)	47.37 (24.45–71.14)	1.33 (0.95–1.72)
*Myotis dasycneme*	7	1	2	1 (1)	14.29 (0.36–57.87)	2 (NA)
Total	5449	2478	6102	10 ( −)	45.47 (44.15–46.8)	2.46 (2.37–2.56)

In general, more female bats hosted bat flies (1251 infested from 2779 sampled, mean prevalence 45.01%, CI 43.15–46.88, mean intensity 2.56, CI 2.42–2.70), than males (1220 infested from 2981 sampled, prevalence 40.92%, CI 39.15–42.71, intensity 2.35, CI 2.22–2.47, Table 2.), with significant differences noted in the prevalence (Fisher`s Exact Test, *p* < 0.01), but not in mean intensity (Mann–Whitney U test, *p* > 0.68).

**Table 2:** Sexual differences in prevalence and intensity of bat fly infestation.

Bat species	Female			Male		
	*n*	Prevalence (%)	Mean intensity	*n*	Prevalence (%)	Mean intensity
*Miniopterus schreibersii*	995	62.21	2.33	1113	66.04	2.21
*Rhinolophus ferrumequinum*	306	21.9	1.37	194	25.26	2.1
*Myotis blythii*	261	66.67	2.26	127	40.16	2.02
*Myotis myotis*	212	67.45	3.07	101	50.5	2
*Myotis daubentonii*	58	32.76	2.84	208	49.52	2.25
*Myotis emarginatus*	148	2.03	2.33	100	1	2
*Rhinolophus euryale*	134	56.72	2.55	111	57.66	2.36
*Myotis capaccinii*	114	80.7	5.28	84	69.05	5.53
*Myotis nattereri*	53	11.32	2.17	104	17.31	1.83
*Rhinolophus hipposideros*	94	3.19	1	56	1.79	1
*Rhinolophus mehelyi*	52	38.46	1.6	72	31.94	1.74

We found significant differences between male and female hosts, in the prevalence of infestation in case of three bat species. Females had higher prevalence in the case of *My. myotis* (Fisher`s Exact Test, p < 0.01) and *My. blythii* (Fisher`s Exact Test, p < 0.001), but males had significantly higher prevalence in case of *My. daubentonii*. No difference was noted between the respective prevalence values of other bat species’ sexes. Intensity of infestation differed significantly among sexes only in the case of* Rhinolophus ferrumequinum*, where males had significantly higher mean infestation values (Mann–Whitney U test, *p* < 0.014), while in case of *My. myotis*, females had significantly higher mean intensity (Mann–Whitney U test, *p* < 0.024, Table 2.).

The highest levels of prevalence (75.8%, CI 69.32–81.65) were found in *My. capaccinii,* followed by *Miniopterus schreibersii* (64.2%, CI 62.07–66.21) and *My. myotis* (62.3%, CI 56.75–67.7). Highest intensity value was recorded also in the case of *My. capaccinii* (5.36 mean intensity, CI 4.59–6.02, n = 169, range 1–29, see also Table 1). In the case of *My. capaccinii* both prevalence (Fisher`s Exact Test, *p* < 0.01) and the mean intensity (Mann–Whitney U test, *p* < 0.05) of infestation were significantly higher than in any other bat species.

### Seasonality of parasitism

**Figure 1:**
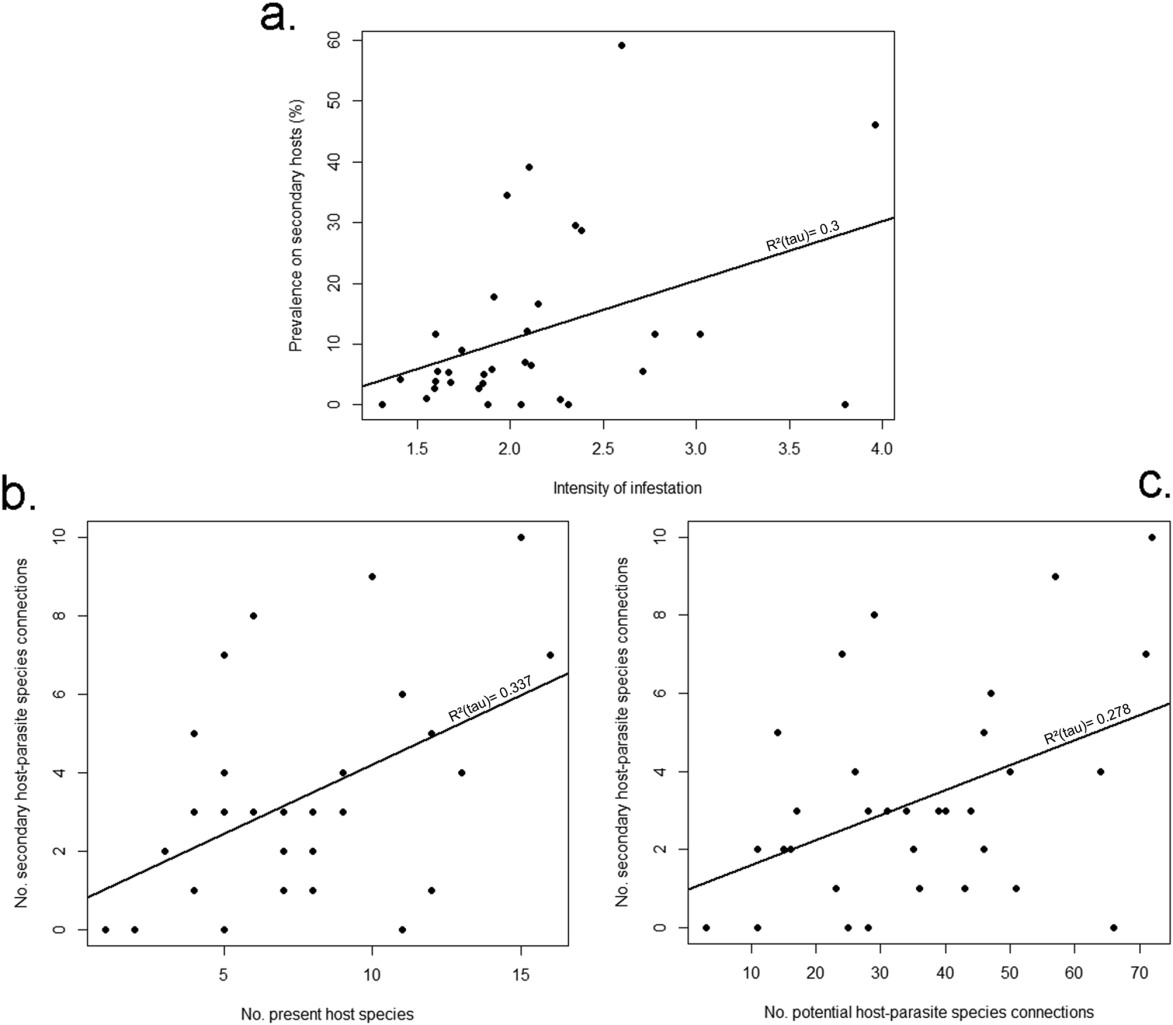
Correlations between the prevalence of bat flies on non-primary hosts and: intensity of parasitism (**a**), the numbers of bat species present at individual roost (**b**), and potential host-parasite connections (**c**).

Bat fly prevalence and mean intensity significantly differed between the two seasons analyzed. Less bats were parasitized in spring than in autumn, with spring prevalence of 41.74% (CI 39.3–44.21) vs. autumn prevalence of 48.91% (CI 47.24–50.59; Fisher`s Exact Test, p < 0.001, Fig. 1). Similarly, mean intensity values were higher in autumn, too. These differences were also significant, with a mean intensity of 2.14 (CI 1.97–2.31) in spring vs. 2.49 (CI 2.38–2.6) in autumn (Mann–Whitney U test, p < 0.001). We found a sexual bias in seasonal infestation patterns, too. In case of males both prevalence and intensity differed significantly. In spring 32.34% (CI 28.41–36.47) of males were infested, but this was 48.36% in autumn (CI 46.19–50.52; Fisher`s Exact Test, p < 0.001). Also, males had a lower mean intensity (1.71, CI 1.53–1.9) in spring, than in autumn (2.38, CI 2.25–2.52, Mann–Whitney U test, p < 0.001). For female bats there was no significant seasonal difference in prevalence (spring 46.57%, CI 43.57–49.64 vs. autumn 49.71%, CI 47.08–52.35), while mean intensity being significantly higher in autumn (2.29, CI 2.07–2.51 in spring vs. 2.64, CI 2.47–2.81; Mann–Whitney U test, p = 0.005, see also Table 3).

**Table 3:** Seasonal differences in the prevalence and intensity of bat fly infestation of individual bat species.

Bat species	Prevalence (%)			Intensity		
	Spring	Autumn	Fisher`s Exact Test	Spring	Autumn	Mann–Whitney U test
*Miniopterus schreibersii* (846/1261)	50.69	72.5	p < 0.001	1.8	2.37	p < 0.001
*Myotis blythii* (159/228)	67.31	51.33	p < 0.01	2.32	2.06	
*Rhinolophus ferrumequinum* (117/385)	13.21	29.59	p < 0.001	1.57	1.7	
*Myotis daubentonii* (77/190)	27.63	56.14	p < 0.001	1.52	2.54	p < 0.01
*Rhinolophus hipposideros* (64/87)	3.13	2.5		1	1	
*Myotis myotis* (73/243)	79.25	55.22	p < 0.01	3.05	2.22	p < 0.01
*Rhinolophus mehelyi* (52/72)	21.15	44.29	p < 0.01	1.27	1.84	p < 0.01
*Rhinolophus euryale* (51/196)	66.67	54.59		2.21	2.55	

Sample sizes in brackets after host’s name (spring/autumn).

### Host specificity of bat flies

**Figure 2:**
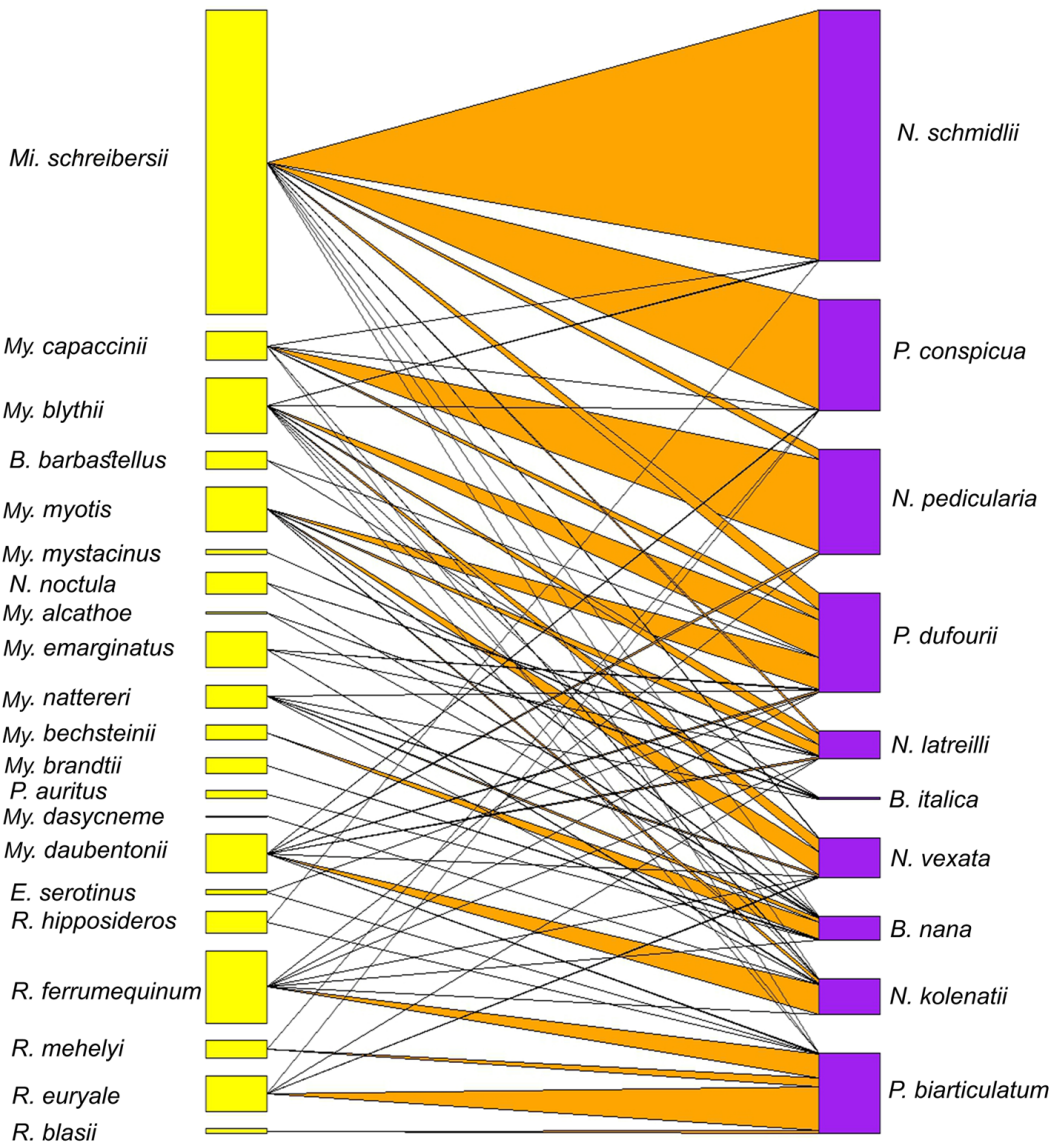
Bipartite representation of the parasite network of bats and bat flies using a quantitative interaction web based on individual host-parasite relations. Links between nodes represent the sum of individual bat fly occurrences for a given bat and bat fly species couple (*yellow* bars—bat species, *purple* bars—bat fly species, *orange* triangles—host-parasite links).

The most widespread bat flies were *Penicillidia dufourii* (found mostly on *Myotis* spp.) and *Phthiridium biarticulatum* (present mostly on bats of the genus *Rhinolophus*, see also Fig. 2). Each of these two parasites was recorded on 7 different host species and at each of the sampling sites (Table 4).

**Table 4:** Main characteristics of bat fly parasitism according to fly species and host-type (primary/non-primary).

Bat fly species	*n*	No. host species	Overall prevalence (%)	Mean intensity	Prevalence on primary hosts (%)	Mean intensity on primary host	Bat flies on non-primary host (%)	Prevalence on non-primary hosts (%)	Mean intensity on non-primary hosts	No. potential parasite-host connections (realized non-primary connections)	No. localities with bat fly presence on a non-primary host, without realized presence on primary host
*Basilia italica*	18	4	4.23	1.29	7.26	1.31	5.56	0.66	1	28 (2)	1
*Basilia nana*	189	10	2.35	2.05	29.01	2.21	11.11	0.44	1.31	75 (5)	3
*Nycteribia kolenatii*	249	9	2.81	2.11	39.47	2.19	7.63	0.33	1.46	96 (4)	0
*Nycteribia latreillii*	246	8	2.84	2.1	14.04	2.13	14.23	0.53	1.94	111 (10)	3
*Nycteribia pedicularia*	826	4	5.1	5.26	66.33	5.48	12.35	0.87	4.08	76 (8)	0
*Nycteribia schmidlii*	1975	7	25.12	2.25	41.15	2.26	0.46	0.58	1.13	106 (6)	1
*Nycteribia vexata*	307	7	3.53	2.18	18.58	2.19	6.51	0.3	2	95 (3)	0
*Phtiridium biarticulatum*	634	10	7.65	2.05	33.33	2.06	1.74	0.25	1.38	129 (24)	1
*Penicillidia conspicua*	876	6	18.55	1.42	28.84	1.42	1.26	0.82	1.1	94 (4)	0
*Penicillidia dufourii*	782	11	10.75	1.66	39.38	1.75	20.46	3.31	1.39	177 (33)	7
TOTAL	6102	22					6.81			–	–

Eight bat species (*R. blasii, R. euryale, R. ferrumequinum, R. mehelyi, My. alcathoe, My. bechsteinii, My. daubentonii* and *My. nattereri*) were primary host for a single bat fly species. Two bat species (*Mi. schreibersii* and *My. cappaccinii*) were primary hosts for two flies, while other two host species (*My. myotis* and *My. blythii*) were primary hosts for three bat fly species (Table 5, on-line Supplementary Table S3). We found no difference in non-primary parasite-occurrence among bats caught in the first hour versus the last two hours of any given capture trial (1.07:0.93, n_first_ = 307, n_last_ = 288), thus we consider that our dataset mirrors real-life situation and is not biased by accidental host-switches due to capture or handling.

**Table 5:** Bat species studied and their respective bat fly species collected in this study (the numbers in the parentheses represent the number of bat flies of the given species recorded on the host species).

Bat species	Primary	Secondary
*Barbastella barbastellus*	*Basilia italica* (3)	*Penicillidia dufourii* (2)
*Eptesicus serotinus*		*Penicillidia dufourii* (1)
		*Phthiridium biarticulatum* (1)
*Miniopterus schreibersii*	*Nycteribia schmidlii* (1791)	*Basilia nana* (1) *Nycteribia kolenatii* (3) *Nycteribia latreillii* (13) *Nycteribia pedicularia* (81) *Phthiridium biarticulatum* (1) *Penicillidia dufourii* (123) *Nycteribia vexata* (1)
	*Penicillidia conspicua* (838)	
*Myotis alcathoe*	*Basilia italica* (6)	*Basilia nana* (3)
*Myotis bechsteinii*	*Basilia nana* (128)	
*Myotis blythii*	*Nycteribia latreillii* (82) *Nycteribia vexata* (107) *Penicillidia dufourii* (285)	*Basilia nana* (2) *Nycteribia kolenatii* (2) *Nycteribia schmidlii* (2) *Phthiridium biarticulatum* (1) *Penicillidia conspicua* (2)
*Myotis brandtii*		*Basilia nana* (2)
*Myotis capaccinii*	*Nycteribia pedicularia* (724) *Penicillidia dufourii* (76)	*Nycteribia schmidlii* (1) *Phthiridium biarticulatum* (1) *Nycteribia kolenatii* (3) *Penicillidia conspicua* (4)
*Myotis dasycneme*		*Nycteribia kolenatii* (2)
*Myotis daubentonii*	*Nycteribia kolenatii* (220)	*Nycteribia latreillii* (11) *Nycteribia pedicularia* (20) *Nycteribia vexata* (3) *Phthiridium biarticulatum* (4) *Penicillidia conspicua* (2) *Penicillidia dufourii* (16)
*Myotis emarginatus*		Nycteribia kolenatii (4) Nycteribia latreillii (3) Penicillidia dufourii (1)
*Myotis myotis*	*Nycteribia latreillii* (59) *Nycteribia vexata* (65) *Penicillidia dufourii* (148)	*Basilia nana* (4)
*Myotis mystacinus*	*Basilia italica* (1)	
*Myotis nattereri*	*Basilia nana* (33)	*Basilia italica* (1) *Nycteribia kolenatii* (1) *Nycteribia vexata* (10) *Penicillidia dufourii* (1)
*Nyctalus noctula*		*Basilia nana* (1) *Nycteribia latreillii* (4)
*Pipistrellus kuhlii*		
*Plecotus auritus*		*Basilia nana* (1)
*Plecotus austriacus*		*Nycteribia schmidlii* (1)
*Rhinolophus blasii*	*Phthiridium biarticulatum* (25)	
*Rhinolophus euryale*	*Phthiridium biarticulatum* (342)	*Nycteribia latreillii* (1) *Nycteribia vexata* (4) *Penicillidia conspicua* (1)
*Rhinolophus ferrumequinum*	*Phthiridium biarticulatum* (182)	*Basilia nana* (1) *Nycteribia kolenatii* (1) *Nycteribia pedicularia* (1) *Nycteribia schmidlii* (3) *Nycteribia latreillii* (1) *Nycteribia vexata* (1) *Penicillidia dufourii* (2)
*Rhinolophus hipposideros*		*Phthiridium biarticulatum* (3) *Nycteribia schmidlii* (1)
*Rhinolophus mehelyi*	*Phthiridium biarticulatum* (69)	*Penicillidia conspicua* (2)

We have found a significant correlation between the intensity of infestation on primary hosts and the prevalence of bat flies on non-primary hosts (on-line Supplementary Table S3, Fig. 1a., T = 0.31, p = 0.014). Also, significant correlation was found between the number of host species present at any given roost and the prevalence on non-primary hosts recorded at that particular roost (see also Fig. 1b, T = 0.33, p = 0.011). There was a significant positive correlation between the number of potential parasite-host connections of hosts present at any given roost and the number of non-primary parasite-host connections for any given fly species, too (Fig. 1c, T = 0.27, p = 0.031). Males had significantly more non-primary parasites than females in term of prevalence (Fisher`s Exact Test, p < 0.001), however, this relationship was detected only in autumn.

The host specificity index (SI) was 96.79% overall; ranging between 92.85% (*Ny. vexata*) and 100% (*B. nana*). This index was higher in spring (97.6%) in comparison to autumn (90.6%), a difference close to significance (Fisher`s Exact Test, *p* = 0.056).

## Discussion

In this study we evaluated the specific relationship bats have with one of their most abundant ectoparasites, the bat fly family Nycteribiidae. Using wild caught bats in the south-eastern region of Europe and collecting their parasites we assessed the host selection of bat flies and their on-host seasonal distribution. All bat fly species previously described from the region^29^ were collected, and we sampled nearly each of the bat species occurring in the region^30^. Probability sampling of bat flies was achieved for most host species (74% of all) and sampling results enabled us to test for two hypotheses using fine-scale analyses of host-parasite relationships of bats and their dipteran parasites.

The overall infestation prevalence of bats with bat flies showed large scale differences between species and seasons. Low parasite prevalence values (< 5%) were recorded for eight bat species. These host species are mainly forest-dwelling, crevice or tree-hole specialists, which roost solitarily or in small groups and frequently change their respective roosting sites, thus avoiding parasite build-up^31,32^. Although these species were sampled both near their maternity roosts (nurseries are made up by significantly larger groups), as well at underground roosts (in the autumn swarming period), their parasite prevalence levels are an order of magnitude lower than most bat species which regularly roost inside underground roosts. Most forest dwelling bat species showed low prevalence and intensity of bat fly infestation, with nine bat species being void of any fly. The two notable exceptions are the Alcathoe bat *My. alcathoe* and the Bechstein’s bat *My. bechsteinii*. Bechstein’s bats are notorious to regularly host their specific bat fly (*B. nana*), likely due to their high roost fidelity habits (recurrent use of robust roosts is the norm for this species, in contrast to most other forest dwellers, which use more ephemeral roosts)^20,33^. However, we have no explanation for the apparent high parasitism rate of Alcathoe bats, especially that they harbored several fly species (4).

We found high levels of bat fly-parasitism (both in terms of prevalence, as intensity) among bat species regularly using underground habitats in the active season. Among these, cave-specialist bats (species permanently residing inside underground roosts) showed the highest prevalence and intensity values (i.e. *Mi. schreibersii* and *My. capaccinii*). This is likely caused by the reduced chance of hosts to avoid colonizing efforts of emerging bat flies. As many bat fly females deposit/lay their 3^rd^ instar larvae close to bottleneck sections of caves (narrowest passages close to entrance, overhangs, etc.), freshly emerging flies have easy access to hosts during the hosts’ daily commuting, when the bats are forced to fly close to these areas^14^ (and also pers. obs. of authors). Moreover, cave-dwelling bats show high fidelity to their roosts^31,31^, in contrast to forest dwelling species, which are known to regularly switch roosts in order to avoid colonization with newly emerging bat flies^20,33,34^.

### Seasonality of parasitism

**Figure 3:**
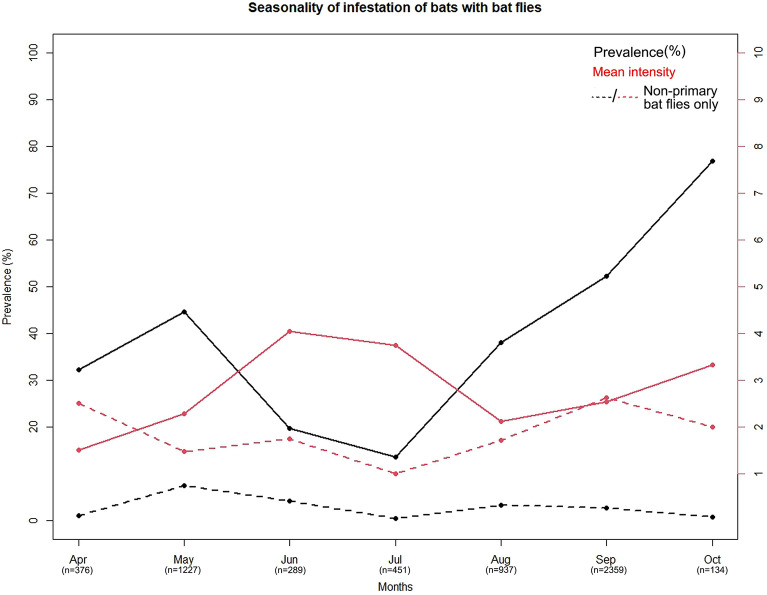
Seasonal dynamics of bat fly prevalence and intensity recorded on bats captured in Bulgaria and Romania. *Black line* – monthly variation of mean prevalence, *red line* – monthly variation of mean intensity.

We found that bat fly infestation shows seasonal fluctuations, significantly increasing towards the end of the active season of hosts (in the study region all bat species hibernate for several months, Ref.^1^). Most bat species (and individuals) had considerably larger ratio of the individuals infested in autumn, with peaking mean intensity also registered in this season (Fig. 3, Table 3). Studies of individual bat species and their respective bat flies already reported similar findings^16,25^. Lourenço & Palmeirim (2008)^25^ found that the reproductive season of the hosts is the main factor which regulates the reproduction of bat flies in the case of the common bent-wing bat (*Mi. schreibersii*), thus bat fly populations synchronize their reproductive output with the hosts’ reproduction^25^. The large groups of nursing females and their young make easy targets to exploit because their reproductive activity requires high effort. Milk-production and breast feeding are highly demanding in terms of energy and time invested, Ref.^35^ and most females are not able to invest the same level of energy in grooming or avoidance, while also showing lower levels of cellular and innate immunity in this period^36^. In consequence, the largest prevalence and intensity of bat flies is recorded in the post-reproductive period (e.g. boreal autumn, see also^16^). A very similar situation was observed in the case of another obligate parasite group, the *Spinturnix* mites, which showed increased prevalence and intensity on females in the mating season^37^. This causes natural annual cycles in bat fly populations in temperate regions, with increasing numbers during the active period of the hosts and decreasing during wintering because of the limited resource availability^5^. This trend was observed in the case of bat fly populations from our study, too (see Fig. 3).

### Host specificity of bat flies

Bat flies are considered highly host specific by most authors^4,13,15,38^, with certain authors explaining occasional occurrence of bat flies on non-primary hosts as accidental, an artefact caused by collection bias^4^ or disturbance transfer during sampling^15^. As our intention was to investigate the host specificity of bat flies in a natural environment, by studying multi-host groups in roosting sites, we took specific precaution measures to avoid such factors. In contrast to most previous assumptions regarding the host specificity of bat flies^15^, we found fairly high degrees of bat fly parasitism on non-primary hosts, especially in the season with high abundance of parasites (boreal autumn). Our results only partially proved the findings of previous studies^5,8^ extending the list of primary hosts for several bat fly species, like *Ph. biarticulatum,* for which all previously listed primary hosts (*R. ferrumequinum* and *R. hipposideros*) were found to be less parasitized than non-primary hosts (*R. blasii* and *R. euryale*).

The least host-specific bat fly species in our study was *Pe. dufourii,* a species which had both the highest number of parasite-host connections (7) and cumulative number of non-primary connections (6) from all locations and bat species. While these results are in concordance with previous studies in the region^8–10^, in our study *Ph. biarticulatum* formed a third category alone, with 88.5% of the flies collected from hosts assumed to be of non-primary-type by the literature^5,8^. This surprising finding is most probably due to the lack of detailed data from this species in the South-East European region, where the supposedly non-primary hosts (*R. euryale, R. mehelyi* and *R. blasii*) are most abundant.

Despite the limited information on the role of competition on host selection at the infracommunity level, it is widely accepted that co-occurrence of multiple fly species on specific host individuals is not a determining factor^39^. In contrast, we found that in bat communities, where the overall bat fly intensity was higher, more flies chose to parasitize individual hosts from non-primary host species, indicating the importance of intraspecific inference competition (Fig. 2.) and also signaling the density-dependence of this trait. In an experiment Dick and Dick (2006)^40^ reached similar results, proving that bat flies offered free choice preferred hosts without conspecifics, however the authors found no difference in host choice if the host was already infested with a different bat fly species.

Our results show that the local presence of multi-species community of hosts favor the occurrence of bat flies on non-primary hosts. This was more common in autumn season when both prevalence and intensity is usually higher on primary hosts, but was observed even on bat species where bat fly abundance showed no difference in comparison to spring (e.g. *My. myotis*, *My. blythii*). Thus reinforcing the hypothesis that host-choice is influenced by the availability of multiple host species and not only by interference competition between flies^41^. Although a higher occurrence of bat flies on non-target hosts may be caused by a mere accidental outcome of host switching, it also may represent an adaptive choice for phoresy, too^42^. Different host species may use different individual roosts in consecutive nights due to high mobility in the autumn caused by active mate searching in host populations during the ‘swarming period’^1^. This temporally occurring, highly promiscuous use of roosts by these (especially male) hosts may allow parasites to disperse between colonies, increasing their choice to locate and colonize new host populations^37^. This second hypothesis is supported also by the bat flies’ preference for choosing male hosts over females in this period (males had significantly higher non-primary bat fly-prevalence than females in autumn, and they showed higher overall intensity of parasitism in this season, too). Male bats are the host gender which has a higher mobility and is more promiscuous in roost selection in the mating period^43–45^. By preferentially choosing male hosts, bat flies may increase the likeness of colonizing new host populations by reaching new roosts in consecutive visits by the swarming males. The use of a non-primary/secondary host species for dispersal and colonization was already suspected for the bat-ectoparasite system in a temperate bat species, but for a different ectoparasite category, the spinturnicid mites^37^.

In conclusion we suggest that the host specificity and host choice of bat fly species are not intrinsic characteristics of bat flies, but may show changes during the active period of the host. Several factors may influence it, like seasonal differences in hosts’ behavior and ecology, by the intensity of infestation of individual hosts’ and the species composition of the local host community (i.e. the absence or presence of multiple non-primary hosts in a particular roost). Choosing non-primary hosts in the mating period of temperate bats may be an adaptive choice for bat flies, thus increasing the dispersive ability of individual bat fly species.

## Methods

### Field work and sample collection

Bat flies were collected from live bats trapped in the spring (March–May) and the autumn (July–October) during 2015–2022. For sampling we selected roosts with large populations of bats, while also targeting roosts along a gradient of species diversity of bats (e.g. from low number of possible hosts—three species in case of three sites, to high number of potential hosts—15 in case of Canaraua Fetii and Peștera Mare de la Merești, see also Online supplementary file S1., with all the sampling sites, bat host species and bat fly species number). Both natural (caves, n = 33, 51.5%), as well artificial (mostly mine shafts, n = 15, 23.4%) underground sites were sampled, together with anthropic roosts, too (buildings, n = 16, 25.0%, see also on-line Supplementary file S1.). Most roosts were sampled multiple times (average 2.75, range 1–12 times). As most roosts (51 out of 64, 79.7%) have multiple entrances, bats were captured close to only one entrance, to reduce overall disturbance. For capturing we used mist nets (D15 mesh, 5 shelf type, 3 to 12 m long, Ecotone Inc.) and harp traps (4 wall, 1.6 × 1.6 m, custom built), set close to the entrances of roosting sites. Bats were extracted from nets or harp trap as soon as they entered and were kept in individual cotton bags until processed. Special care was taken at the moment of extraction to avoid any possible accidental cross-contamination with bat flies from neighboring individuals. In addition, any bag was used only once at any capture trial/location and washed after each use. Only bats actively emerging from the roosts were targeted, and only apparently healthy bats were sampled. No sampling occurred inside roosts. During their examination, the species, age and sex of the hosts was noted, with forearm length (mm) and mass (g) established for each individual. The identification of bats was based on morphology^1^. Bat flies were removed individually with a forceps and stored in ethanol, in individual vials for each host. All visible parasites were collected. A bat was considered bat fly-free, if no additional fly was observed after the host was inspected twice all over its body by blowing its fur (bat flies start moving on the host if disturbed, see Ref.^13^).

Bat flies were identified (species and sex) in laboratory under binocular microscope (Olympus BX61 microscope, Olympus Corporation, Tokyo, Japan), using morphological keys^5,46^. For host specificity calculations, basic specificity (number of host species) of each fly species for each site was determined. Bat flies on their main host are frequent and abundant and they occasionally use non-primary hosts in fewer numbers. Bat species were assigned as *primary hosts* using the 5% threshold rule (a bat species was considered the primary host of the respective bat fly species, if more than 5% of all the bat flies’ records were registered on individuals of the respective host species^15^. Any bat species hosting less than 5% of all individual occurrences was assigned the *non-primary host* status. Bat flies were characterized as *monoxeonous*, *oligoxenous* or *polyxenous*, according to the number of primary hosts^9,15^. We calculated the *potential host-parasite connections* (number of potential host species for each individual bat fly species present at any given location, using the list of potential connections obtained from literature^8^) and the *realized host-parasite connections* (number of host-parasite connections between any given bat fly and its host species recorded at each sampling location). We controlled for potential sampling bias (accidental cross-contamination due to handling of hosts) using the ratio of accidental hosts in the beginning of capture (first hour) and end of capture last two hours (we used two hours at the end, as number of captures are higher in the beginning). A ratio close to 1:1 was considered as natural/random^13^.

Bats were handled according to the current law of animal welfare regulation (L206/2004), and the Research Bioethics Commission of USAMV CN approved the used methodology of bat handling. Permission from the Institutional Animal Care and Use Committee (IACUC) was not necessary, because bats were released in the field immediately after bat fly removal (none taken to participating Institutes). Bat capture licenses were issued to ADS and are the following: 305/2015, 46/2016, 24/2017, 111/2018, 103/2019, 81/2021 and 122/2022.

### Statistical analyses

Data were statistically analyzed using the R software (Version 3.2.3)^47^. Mean prevalence (percentage of the infested bat host individuals from any population) and mean intensity (mean number of parasites on the infested individuals) values were calculated for each host and parasite species independently. For each bat fly species we calculated the specificity index (SI), the percentage of total bat flies of a single species found on the main host^48^. We compared seasonal differences (two seasons, *spring*—before maternity period, March to mid-May; *autumn*—after maternity period, July–October) in prevalence, intensity and SI for all host and parasite species, where large enough sample size was recorded (min. 50 host/season, i.e. spring or autumn, data collated from multiple sites for both seasons). Prevalence and SI values were compared statistically by Fisher’s exact test and intensity values by Mann–Whitney U tests. To test for differences in sampling effort between seasons, we compared the host species composition (number of species present at individual roost, as well the diversity of captures using *Shannon*–*Wiener* index), capture efficiency (number of average captures/collecting trial) and bat fly prevalence of sampled roosts. We used Bonferroni post hoc tests to compare differences among means when a significant treatment effect was found. Due to non-normal distribution of data we used Kendall’s rank correlation Tau to investigate dependence between variables^49^. All tests were considered significant when *p* < 0.05.

### Ethics approval and consent to participate

Permission for bat capture was provided by the Underground Heritage Commission (Romania) and the Bulgarian Ministry of Environment and Water (permit no. 718/24.08.2017). Bat banding license numbers are 305/2015, 46/2016, 24/2017, 111/2018, 103/2019, 81/2021 and 122/2022. Bats were handled according to the current law of animal welfare regulation (L206/2004), and the Research Bioethics Commission of USAMV CN approved the used methodology of bat handling. Permission from the Institutional Animal Care and Use Committee (IACUC) was not necessary, because bats were released in the field after fly removal (none taken to participating Institutes). No live bat was harmed for this study.

### Supplementary Information

Supplementary Table S1.

Supplementary Table S2.

Supplementary Table S3.

## Data Availability

All data are available in the main text, or the electronic supplementary material.
